# *Streptococcus parasanguinis*: An emerging pathogen causing neonatal endocarditis: A case report

**DOI:** 10.1099/acmi.0.000576.v4

**Published:** 2023-06-29

**Authors:** Twishi Shrimali, Shikhir Malhotra, Nidhi Relhan, Vibhor Tak, Sushil Kumar Choudhary, Neeraj Gupta, Arun Kumarendu Singh

**Affiliations:** ^1^​ Microbiology, All India Institute of Medical Sciences, Jodhpur, Rajasthan; ^2^​ Neonatology, All India Institute of Medical Sciences, Jodhpur, Rajathan

**Keywords:** infective endocarditis, *Streptococcus parasanguinis*, neonatal sepsis

## Abstract

**Background.** The microbial infection of the endocardium, popularly known as Infective Endocarditis (IE), is typically classified on the basis of anatomy, valve nativity and its associated microbiology. As per the associated microbiology, *Staphylococcus aureus* is the most common microorganism responsible for the cause of IE. Even though, the Streptococcus group accounts for a smaller percentage of IE, however this doesn’t give us the liberty of ignoring the high mortality and morbidity associated with this pathogen.

**Case presentation.** We report an unusual case of neonatal sepsis, complicated with endocarditis, caused by penicillin resistant *Streptococcus parasanguinis *. The neonate however died of the same despite all efforts. The said baby was given birth by a mother with gestational diabetes mellitus.

**Conclusion.:**

High index of clinical suspicion and prompt diagnosis are the most important factors of patient management, especially in cases of life threatening neonatal infections. In such conditions a coordinated interdepartmental approach is very much needed.

## Data Summary

No new data, tools, software or code was used for this case report. Supplementary data file reports of VITEK MS (bioMérieux Inc) (data: S1.a, available in the online version of this article) and VITEK 2.0 Compact (bioMérieux Inc) (data: S1.b) by which the identification and antibiotic susceptibility testing of the isolated organism (*Streptococcus parasanguinins*) were performed.

## Introduction

Discovered by Whiley *et al*. 1990, *Streptococcus parasanguinis* is a Gram-positive, non-motile, catalase-negative bacterium. Known to be a coloniser of the human oral cavity, it is primarily associated with formation of dental plaques [1] in the adult population. This microbe can be isolated from human breast milk [2] and from human infant intestine as well [3]. The pathogenicity of this bacterium has been reported in literature in some places, however, the same has not been established yet in the case of neonates. Scarcely *S. parasanguinis* can migrate to the bloodstream, and which could result in infective endocarditis [4–7] and peritonitis [8]. Inappropriate diagnosis of IE may lead to a delay in treatment, resulting in valvular obstructions, sepsis, multi-organ failure and death of the patients. We present a rare and unusual case report of *S. parasanguinis* sepsis in a 1 day old infant and its clinical implications.

## Case presentation

**Fig. 1.**
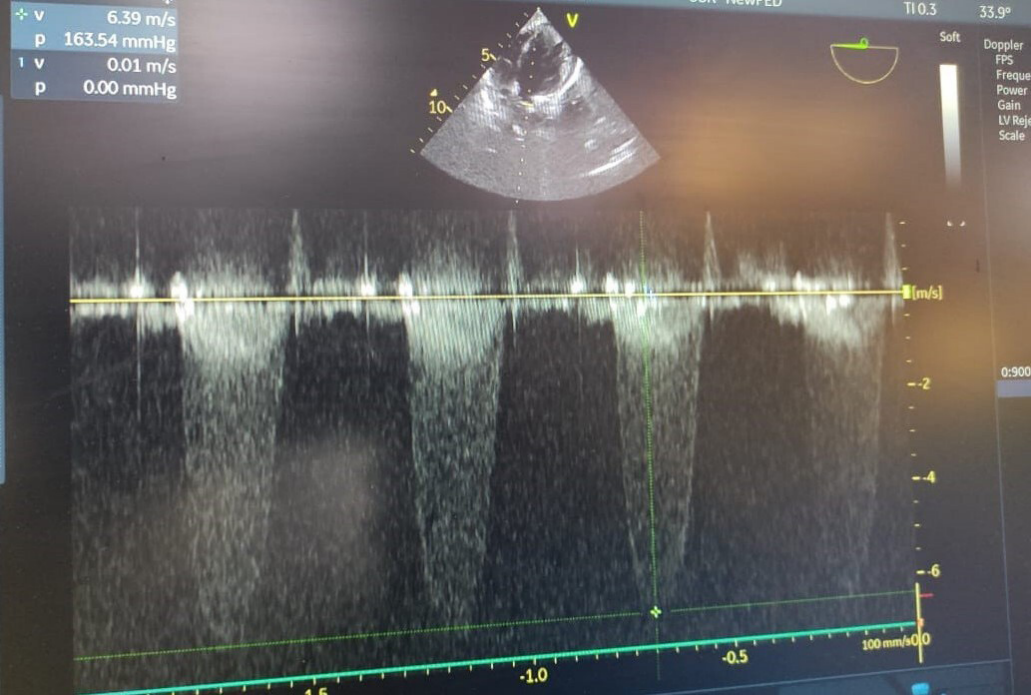
M mode of echocardiograph showing increased gradient across aortic valve of 163.54 mmHg and V max of 6.39 m s^−1^.

**Fig. 2.**
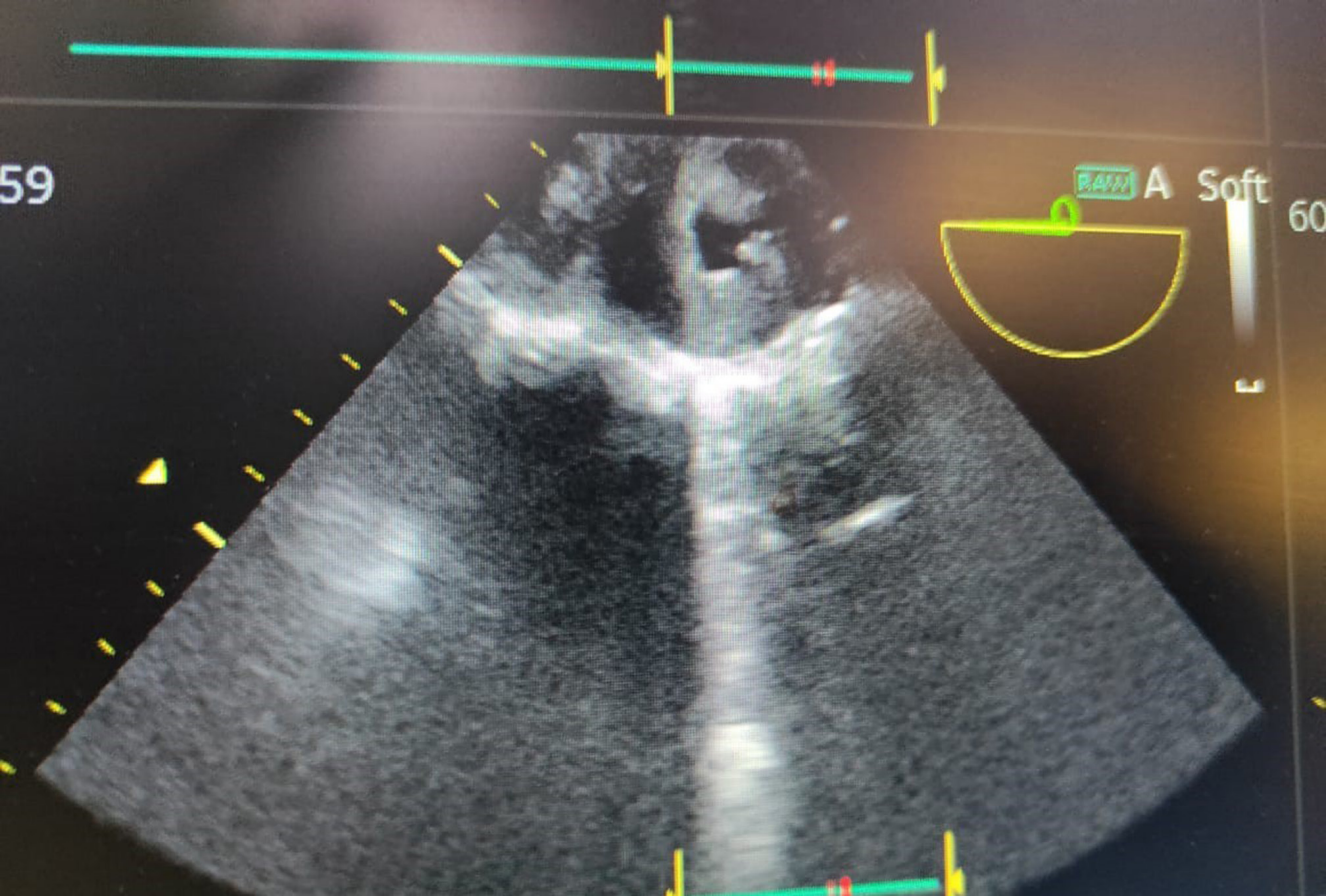
: 2-D transthoracic echo showing severe stenosis and mild regurgitation at the level of aortic valve.

A male child, with appropriate gestational age and born to a 28 years old primigravida with history of gestational diabetes mellitus, was transferred to the neonatal intensive care unit of our institute in July 2022. The neonate had developed respiratory distress with partial pressure of saturated oxygen level of 79 % at 5 h of life and Downe’s score of 3/10. On examination of the baby, he was found to have tachypnoea (65 cycles/min), tachycardia (134 min^−1^), and birth weight of 3408 grams. The child also had peripheral cyanosis with cold extremities. Blood hemogram revealed, haemoglobin of 12.7 g dl^−1^ and total leucocyte count of 15 200 mm^−3^. Sepsis markers indicated that the procalcitonin was 5.4 ng ml^−1^ and C-reactive protein was 26.6 mg l^−1^. In view of the above ailments, possibilities of heart disease was predicted, and a 2-D echocardiography was advised. A 2-D echocardiography revealed the presence of bicuspid aortic valve with critical aortic stenosis, mild aortic regurgitation and persistent pulmonary hypertension (Figs 1 and 2).

The chest ultrasound also indicated the presence of bilateral pleural effusion. Accordingly the baby was immediately started on CPAP support with 21 % fiO_2_ and PEEP of 5 cm water, which were increased to 30 % and 6 cm of water respectively, at around 12 h of life. A diagnosis of respiratory distress with critical aortic stenosis with bicuspid aortic valve with refractory cardiogenic and pulmonary oedema was made. In view of suspected sepsis, blood samples were sent in paediatric BACTEC bottles to the department of Microbiology for aerobic culture and sensitivity. However, the baby was empirically started on piperacillin-tazobactam and amikacin. For persistent pulmonary hypertension, oral sildenafil was also started.

After an interval of 12 h, another blood sample was sent for aerobic culture and sensitivity. The first blood culture bottle flagged positive after 15 h 39 min of aerobic incubation at 37 °C and the second one after 13 h 24 min. Initial Gram-stain from both the flagged bottles revealed uniformly stained Gram-positive cocci aligned in the form of chains under 1000 × magnification. Plating was done from the blood culture bottles on pairs of 5 % sheep blood and chocolate agar. The plates were incubated in CO_2_ and routine aerobic incubator for 16–18 h at 37 °C.

**Fig. 3.**
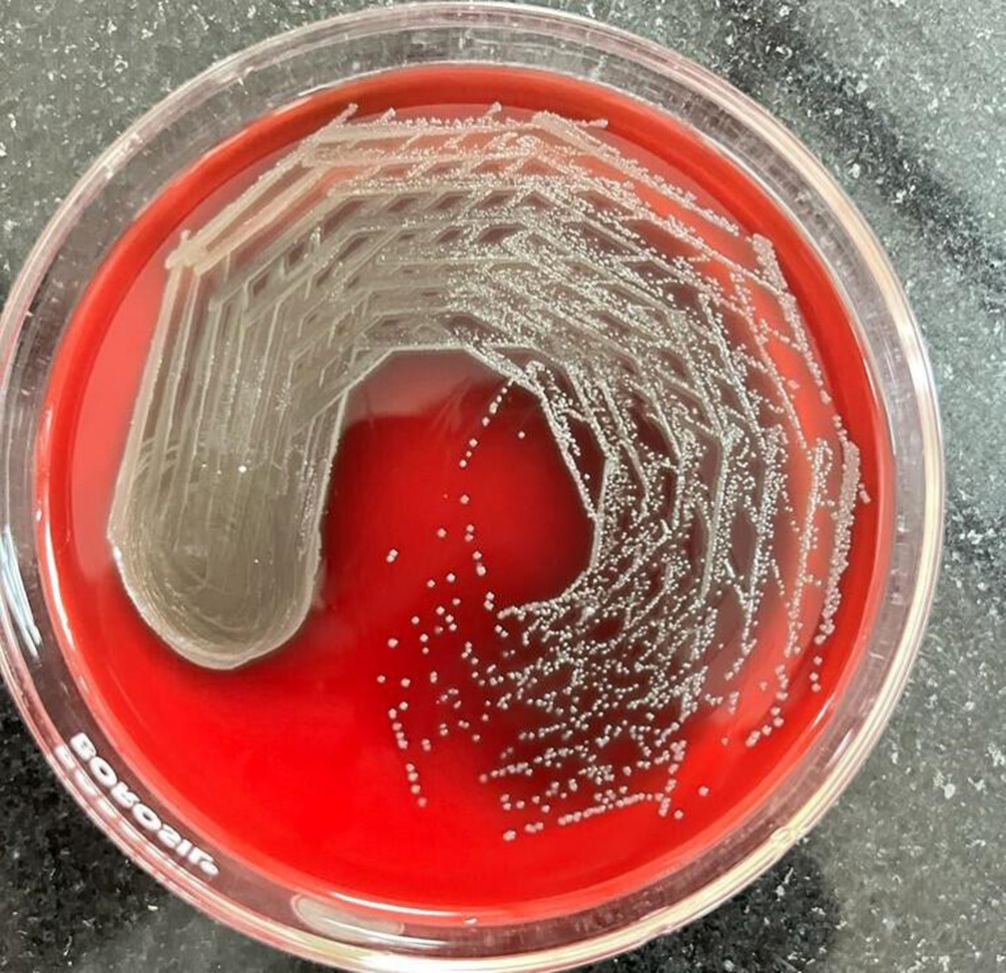
Pin point alpha haemolytic circular low convex semi-transparent smooth low convex colonies on blood agar with 5 % sheep blood.

**Fig. 4.**
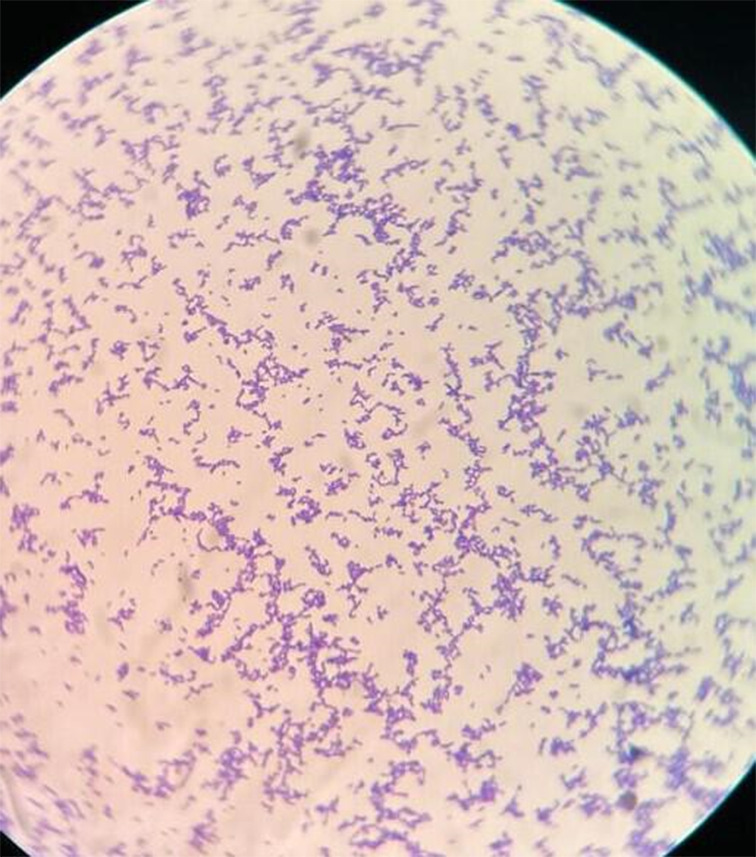
Gram-staining showing Gram-positive uniformly stained cocci seen under 1000× magnification.

Culture growth obtained from both the flagged bottles were identical. The colonies grew to approximately 0.5 mm in size, which were round in shape with irregular margins, flat surface, opaque, easily emulsifiable and showed alpha haemolysis (Fig. 3). Gram-stain from the colonies showed uniformly stained Gram-positive cocci arranged in chains (Fig. 4). The isolated colonies were processed by VITEK MS (bioMérieux Inc) (data: S1.a, Supplementary Material 1) and VITEK 2.0 Compact (bioMérieux Inc) (data: S1.b, Supplementary Material 1), which gave the identification as *Streptococcus parasanguinis* with 99 % probability. The isolate was sensitive to linezolid and vancomycin but resistant to ampicillin, clindamycin, ceftriaxone, tetracycline, cefotaxime, penicillin, moxifloxacin and levofloxacin.

**Fig. 5.**
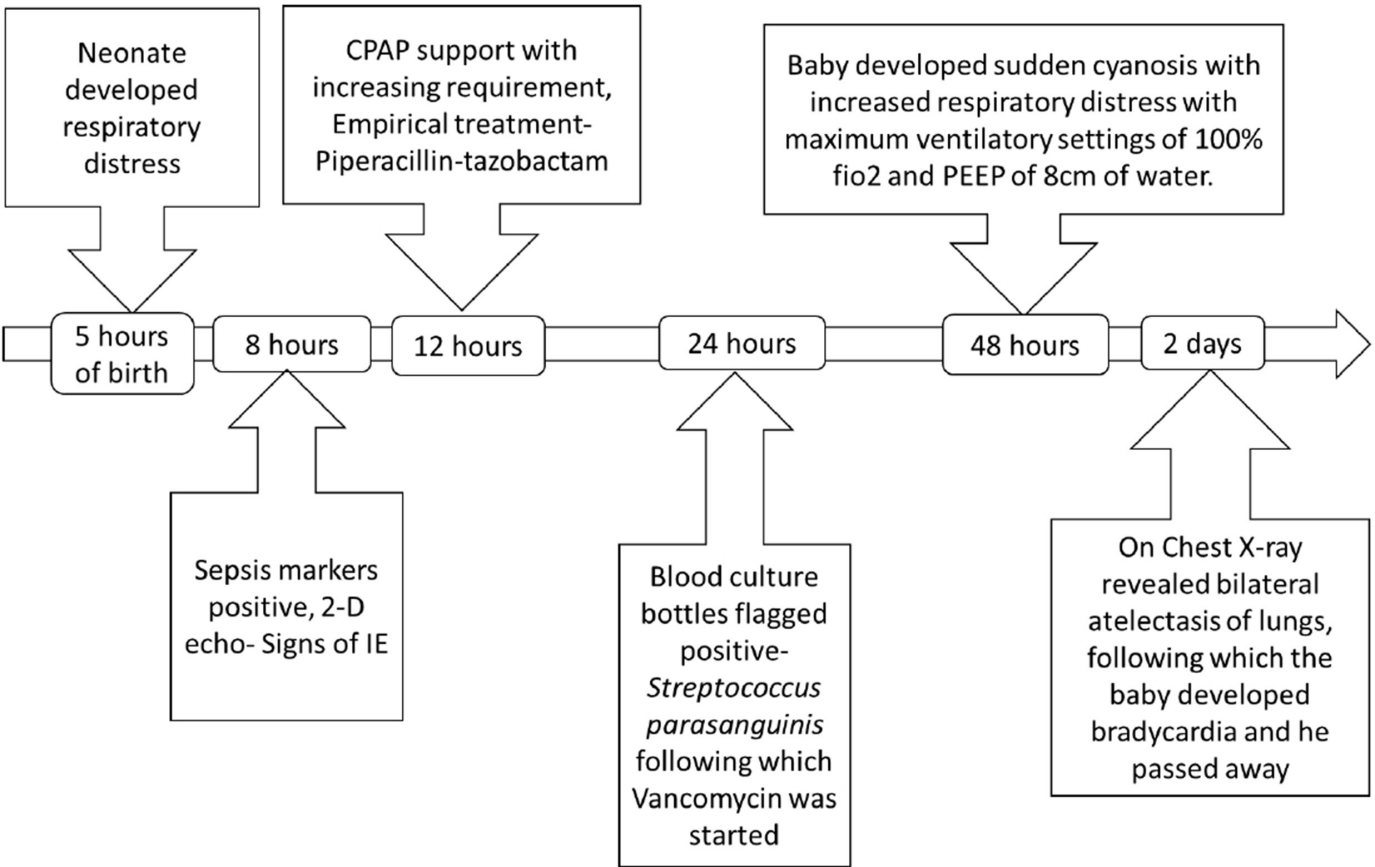
Timeline depicting the series of events.

On the intervening night of the above investigation, baby developed sudden cyanosis with increased respiratory distress with maximum ventilatory settings of 100 % fiO_2_ and PEEP of 8 cm of water. In view of severe hypotension baby was started on inotropic support of dopamine, adrenaline, nor-adrenaline and vasopressin. Chest X-ray revealed bilateral atelectasis of lungs, following which the baby developed bradycardia. Cardiopulmonary resuscitation was started using bag and mask ventilation. Adrenaline and calcium gluconate injections were also given. However, despite of all the efforts, baby could not be revived. Fig. 5 depicts the chronology of events in brief.

## Discussion

Being the pathogenic member of viridans group of streptococci; *S. parasanguinis* has been isolated from oral cavity, upper respiratory tract, gastrointestinal and genitourinary tract [9]. *Streptococcus parasanguinis* may initiate formation of bio-film in newborns when it is present in breast milk. The extra-polysaccharide matrix that makes up bio-films is made up of microcolonies made up of different types of pathogenic bacteria and colonisers. It can be attributed to *S. parasanguinins'* long peritrichous fimbrial structure, which also controls its oral cavity adherence and intracellular resistance to macrophages [10, 11]. This characteristic aids in the formation of vegetations and biofilms, both of which contribute to IE.

Infection of the endocardium occurs when a platelet-fibrin complex adherent to the endothelium gets secondarily infected to produce vegetations, which in turn damages the endocardial tissue and/or valves resulting into stenosis or regurgitation. This disease, which has a death rate of up to 30 % in a month, affects 3–10 individuals per 100 000 population [12]. Because of continual technological breakthroughs, the epidemiology of this ailment has evolved throughout time, making it difficult for doctors to successfully treat it. Furthermore, if we adhere to Western standards, it becomes difficult to manage infective endocarditis in poorer countries where the typical profile is more common. Because India is a developing country, everything in it is changing, including the host factors, vulnerable population, diagnostic methods, antibiotics, and surgical procedures. The situation becomes more complicated as a result of the combined load of traditional and modern obligations, leaving diagnosticians and clinicians perplexed about effective patient care [13]. *Staphylococcus aureus* is currently the most prevalent cause of IE in most studies (26.6 %) followed by viridans group of streptococci (18.7 %). Diagnosis is made considering the predisposing risk factors (cardiac ailment, cardiac intervention, poor socioeconomic status, IV drug abuse, immunosuppression), clinical suspicion (fever, chills, sweating, shortness of breath, loss of appetite and weight, chest pain, peripheral oedema, murmers, haemorrhages, infarcts), findings on echocardiography and blood culture report [12, 14]. All of them lay the foundation for the well-known Duke’s criteria for IE diagnosis. Our patient had severe aortic stenosis with mild aortic regurgitation as reported in the 2D echocardiograph. While the hallmark echocardiographic evidence of IE is a valvular vegetation, other complications involving valvular leaflets (e.g. perforation or pseudoaneurysm), paravalvular structures (e.g. abscess, stenosis, pseudoaneurysm or fistula), or prosthetic valves (e.g. valvular dehiscence) can also be indicative of IE, which can be visualised by transthoracic echocardiography (TTE), as mentioned in the imaging criteria of 2023 Duke-ISCVID Criteria for Infective Endocarditis [15, 16].

Antimicrobial therapy should not be started until three sets of blood cultures are taken which is known to detect bacteraemia in 98 % of cases making blood culture one of the most important investigations to diagnose infective endocarditis [12]. However, in cases of neonatal infections, one should only take one or two blood culture samples in paediatric blood culture bottles, especially if the isolated organism is known to be a typical organism to cause a specific illness. This is to prevent significant blood loss and its accompanying complications [17].

## Conclusion

*Streptococcus* spp. in particular viridans group is frequently isolated in blood culture as pathogenic organisms in case of infective endocarditis and sepsis. Isolation and identification of *S. parasanguinis* in infants are widely underdiagnosed. Authors report a unique case of *S. parasanguinis* sepsis in a 1 day old neonate, a bacterium previously not described as a pathogenic agent for infective endocarditis and sepsis. The key to identify such infection includes a high index of suspicion, diligent clinical examination and a serial paired blood culture sampling. Early diagnosis and prompt therapy can reduce the risk of morbidity and mortality attributed by *S. parsanguinis* infection.

## Supplementary Data

Supplementary material 1
