# Supplementary material for: Streptococcus parasanguinis: An emerging pathogen causing neonatal endocarditis: A case report
Source: Access Microbiol. 2023 Jun 29;5(6):acmi000576.v4. doi: 10.1099/acmi.0.000576.v4 (PMC10323779; doi:10.1099/acmi.0.000576.v4)
Supplement: Supplementary material 1 [file acmi-5-576.v4-s001.pdf]

VITEK® MS Review Detail

❯ Delayed VITEK® MS Review ❯ VITEK® MS Review Detail

Isolate information

Accession ID: 18052022BC582-1 **Specimen Type:** - **Confidence Level:**

Number of identifications: 1

List of identifications

	Position	Analysis Date	Organism Name	Pathogenicity	Confidence Value	Confidence Level	Acquisition/Computation message(s)
	B4	5/18/22 11:56 AM	Streptococcus parasanguinis		99.9		

Key: **Validate selection** **Add comment**

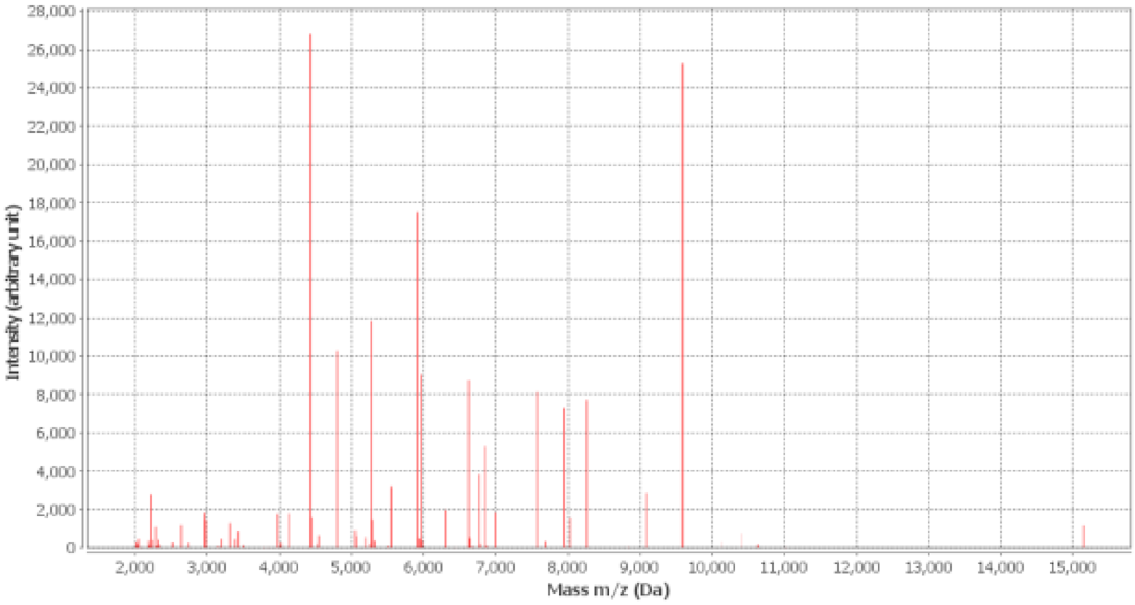

S1.a: Report of VITEK® MS (bioMerieux Inc): Used to identify the isolated organism (*Streptococcus parasanguinis*)

bioMérieux Customer: MICRO

AIIMS JODHPUR
Microbiology Chart Report

Printed May 19, 2022 9:21:25 AM IST

Patient Name:
Location:
Lab ID: 18052022BC582

Patient ID:
Physician:
Isolate Number: 1

Organism Quantity:
Selected Organism : Streptococcus parasanguinis

Source:

Collected:

**Comments:**	Please Check for new intermediate interpretive catagoryintroduced by CLSI 2020that states the names of drugs which has the potential to concentrate at any anatomical site, urine or epithelial lining

Susceptibility Information	**Analysis Time:** 10.93 hours	**Status:** Final

Antimicrobial	MIC	Interpretation	Antimicrobial	MIC	Interpretation
Benzylpenicillin	>= 8	R	Clindamycin	>= 1	R
Ampicillin	>= 16	R	Linezolid	<= 2	S
Cefotaxime	>= 8	R	Teicoplanin		
Ceftriaxone	4	R	Tetracycline	4	I
Gentamicin			Tigecycline	0.5	
Levofloxacin	>= 16	R	Chloramphenicol	2	S
Moxifloxacin	>= 4	R	Rifampicin		
Inducible Clindamycin Resistance			Trimethoprim/ Sulfamethoxazole		
Erythromycin					

AES Findings

**Confidence:**	Consistent with correction

S1.b: Report of VITEK 2.0 Compact (bioMerieux Inc): used to determine the antibiotic susceptibility of the isolated organism (*Streptococcus parasanguinis*)
